# A Reagentless Amperometric Formaldehyde-Selective Chemosensor Based on Platinized Gold Electrodes

**DOI:** 10.3390/ma10050503

**Published:** 2017-05-06

**Authors:** Olha Demkiv, Oleh Smutok, Mykhailo Gonchar, Marina Nisnevitch

**Affiliations:** 1 Department of Analytical Biotechnology, Institute of Cell Biology, Drahomanov Street 14/16, 79005 Lviv, Ukraine; smutok@cellbiol.lviv.ua (O.S.); gonchar@cellbiol.lviv.ua (M.G.); 2 Institute of Applied Biotechnology and Basic Sciences, University of Rzeszow, Kolbuszowa 36-100, Poland; 3 Department of Chemical Engineering, Biotechnology and Materials, Ariel University, Ariel 4070000, Israel

**Keywords:** formaldehyde, amperometric chemosensor, platinized gold electrode

## Abstract

Fabrication and characterization of a new amperometric chemosensor for accurate formaldehyde analysis based on platinized gold electrodes is described. The platinization process was performed electrochemically on the surface of 4 mm gold planar electrodes by both electrolysis and cyclic voltamperometry. The produced electrodes were characterized using scanning electron microscopy and X-ray spectral analysis. Using a low working potential (0.0 V vs. Ag/AgCl) enabled an essential increase in the chemosensor’s selectivity for the target analyte. The sensitivity of the best chemosensor prototype to formaldehyde is uniquely high (28180 A·M^−1^·m^−2^) with a detection limit of 0.05 mM. The chemosensor remained stable over a one-year storage period. The formaldehye-selective chemosensor was tested on samples of commercial preparations. A high correlation was demonstrated between the results obtained by the proposed chemosensor, chemical and enzymatic methods (*R* = 0.998). The developed formaldehyde-selective amperometric chemosensor is very promising for use in industry and research, as well as for environmental control.

## 1. Introduction

Formaldehyde (FA) is a typical indoor air pollutant and is highly toxic to all animals and humans, even at concentrations as low as 0.1 ppm [1,2,3]. According to data of the International Agency for Research on Cancer (IARC) [2,3], FA is a toxic compound that reacts with macromolecules in different biological systems and has mutagenic, immunogenic, allergenic, and carcinogenic effects [4,5,6,7,8]. FA has recently been described as one of the chemical mediators of apoptosis [9]. FA is an extremely toxic agent which is, nonetheless, found in over 2000 commercial products. It is used as an industrial fungicide, germicide, and disinfectant, and as a preservative in mortuaries and medical laboratories. In 2013, the annual FA production was estimated at over 50 million metric tons [6]. Wastewaters from many different industries contain FA and phenols which, in combination, are especially hazardous to living organisms even at low concentrations. Formaldehyde also occurs naturally in the environment. It is produced in small amounts by most living organisms as part of normal metabolic processes. FA is currently considered to be the main cause for the sick building syndrome, which is defined as a set of symptoms associated with irritation of the upper air passages and eyes caused by harmful compounds (particularly FA) which are found in building materials, tobacco smoke, some medicinal preparations, etc. [3,4,5,6,7].

Several analytical approaches for the determination of FA were reported in the last two decades of the 20th century. These methods include spectrophotometry [10,11,12], gas chromatography [13], high-performance liquid chromatography [14], ion chromatography [15], and polarography [16]. These methods require expensive and bulky instruments that have a high power demand and need well-trained operators. They are, therefore, clearly unable to provide real-time information on exposure to FA.

A number of attempts to develop biosensors for the detection of FA were reported, including amperometric sensors [17,18,19,20] and potentiometric detection schemes [21]. FA-selective biosensors are based on cells [18] or enzymes, such as alcohol oxidase (AOX) or formaldehyde dehydrogenase (FdDH), which are used as biorecognition elements [19,20,21,22,23,24,25]. Potentiometric biosensors consisting of a pH-sensitive field effect transistor as a transducer, and containing either the enzyme AOX or permeabilized yeast cells, have been described by Korpan et al. [21]. Conductometric biosensors are based on AOX or FdDH [22,23], and the latter enzyme also serves as a biorecognition element in amperometric biosensors [24,25].

Chemosensors have several advantages for FA analysis over enzyme-based biosensors: Asimple preparation procedure that does not entail the use of expensive enzymes, co-factors, electron transfer mediators and any covering paints or permselective membranes. Moreover, chemosensors are much more stable and their stability depends on the properties of chemocatalysts. Use of metal nanoparticles is a perspective approach to improve the sensors’ characteristics due to their high catalytic ability and low price. In general, the catalytic properties of metals are dependent upon the layer size, composition, and structure, as well as on the support materials [26].

Due to its unique physical and chemical characteristics, including high durability and specific activity, platinum (Pt) stands out from other transition metals in its electrocatalytic efficiency [27,28]. However, the high cost and a limited supply of Pt are limiting factors. The use of nano-scale Pt particles by controlling the morphology and surface structure is, therefore, an effective way for reducing the amount of required Pt and enhancing the electrochemical activity [28,29,30,31,32,33,34,35].

FA electrooxidation on metal surfaces, such as Pt, Cu, Rh, and Pd, has been widely reported [27,36,37,38,39,40,41]. However, some serious drawbacks of FA-selective chemosensor systems, such as low sensitivity and poor selectivity, remain unsolved. These limitations increase the need for the development of new chemosensory systems which will be usable for accurate FA analysis in commercial samples. In the present work, we describe the construction and detailed characterization of new Pt-based chemolayers obtained electrochemically on the surface of gold planar electrodes by both electrolysis and cyclic voltamperometry. The Pt-based chemoelectrodes were tested for analysis of commercial disinfectants and compared to analytical devices reported by us earlier [20,33].

## 2. Results and Discussion

### 2.1. Development of Procedures for Chemocatalyst Formation on the Electrode Surface

**Figure 1:**
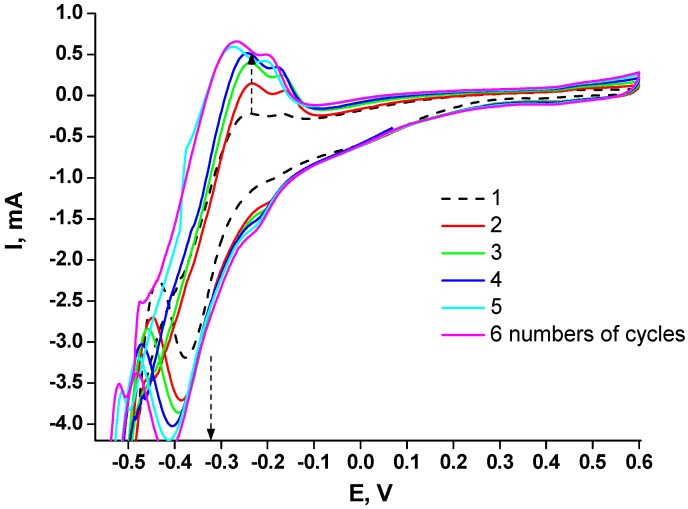
Cyclic voltammograms of Ptelectrodeposition on the surface of 4 mm DRP-C220AT “DropSens”gold planar electrodes. Conditions: −0.6 to 0.6 V vs. Ag/AgCl; scan rate 50 mV·s^−1^.

An additional modification of the electrode surface by Pt layers was used for improving the electrochemical properties of the FA-sensitive chemoelectrodes. For this aim, commercial 4 mm gold planar electrodes DRP-C220AT from DropSens (Llanera, Asturias, Spain) were platinized. Two approaches for this process were applied. The first was based on electrochemical preparation of the surface by electrolysis/H_2_PtCl_6_ in HCl solution according to the method described by Kovalyshyn et al. and by us [20,33]. Electrodes prepared using this method are designated as Pt-1. The second approach included electrodeposition of Pt on DRP-C220AT gold planar electrodes from the same solution using cyclic voltamperometry in the range from −0.6 to 0.6 V vs. Ag/AgCl. Electrodes prepared by the second method are designated as Pt-2. Figure 1 presents cyclic voltammograms of Pt electrodeposition after a number of cycles. It can be seen that each cycle caused improvement in redox properties of the electrode, and the best results were obtained after sixcycles. It should be mentioned that the change after the fifthcycle was very small and the optimal number of electrodeposition cycles of Pt is considered to be about six. Using more than six electrodeposition cycles may cause the formation of a thick Pt-layer which will have a negative effect on the electrode’s electrocatalytic properties.

### 2.2. Determination of Structural and Electrical Properties of the Obtained PtLayers

**Figure 2:**
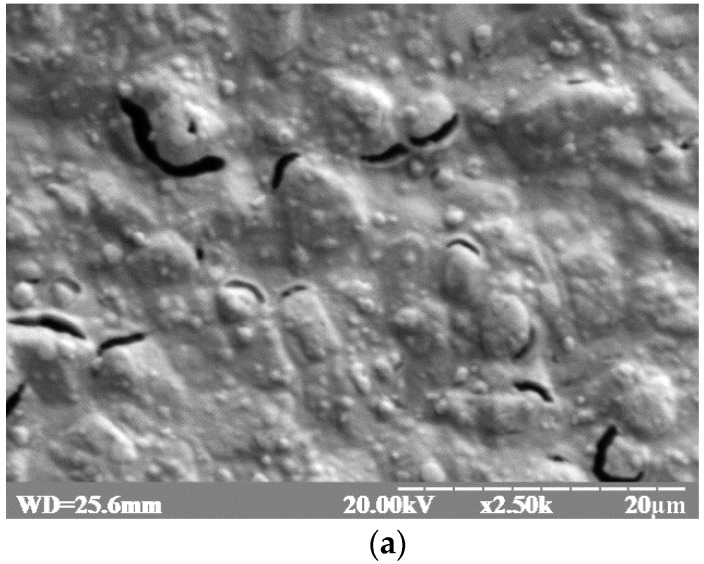

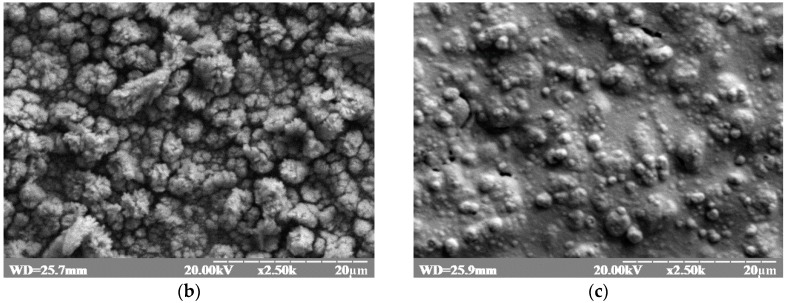
Scanning electron microscopy of (**a**) the surface of a non-modified 4 mm DRP-C220AT “DropSens” gold planar electrode; (**b**) the same surface after modification by Pt using electrolysis (Pt-1); and (**c**) the Ptlayer obtained by electrodeposition (Pt-2). Abbreviations: WD: distance from the last lens of the microscope to the samples (mm); kV: accelerating voltage; x: fold magnification n; μm: scale unit.

The formation of Pt layers on the surface of commercial electrodes was confirmed by scanning electron microscopy using a REMMA-102-02 SEM microanalyzer (SELMI, Sumy, Ukraine). The SEM images demonstrate differences in the surface structure of the non-modified (bare) planar electrode, and the planar Pt-1 and Pt-2 modified electrodes (Figure 2). The non-modified electrode has a smooth surface with very moderate 0.2–4 μm convexities (Figure 2a). The surface of the Pt-1 modified electrode has highly-developed superficies represented by multiple 3–5 μm cone structures (Figure 2b). The surface of the Pt-2 modified electrode appears smoother than the surface of the Pt-1 electrode and has irregular convex 1–5 μm structures (Figure 2c).

**Figure 3:**
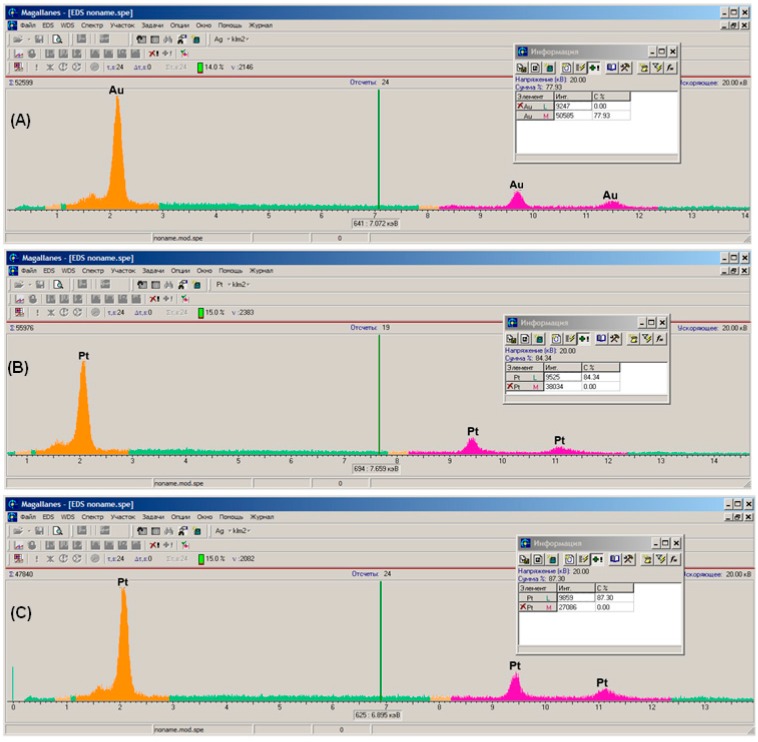
X-ray spectral analysis of (**A**) the surface of a non-modified 4 mm DRP-C220AT “DropSens” gold planar electrode; (**B**) the same surface after modification by Pt using electrolysis (Pt-1); and (**C**) the Ptlayer obtained by electrodeposition (Pt-2).

The formation of different types of Ptlayers was further confirmed by X-ray spectral analysis by interpretation of Kα peaks at 2.1 keV which are characteristic for noble metals (Figure 3). The X-ray spectrogram of the gold electrode before modification represents signals of Au^0^ only, as expected (Figure 3A). However, after modification by the Pt-1 (Figure 3B) and the Pt-2 (Figure 3C) methods, the only registered signal on the surface of the electrodes is related to Pt^0^.

**Figure 4:**
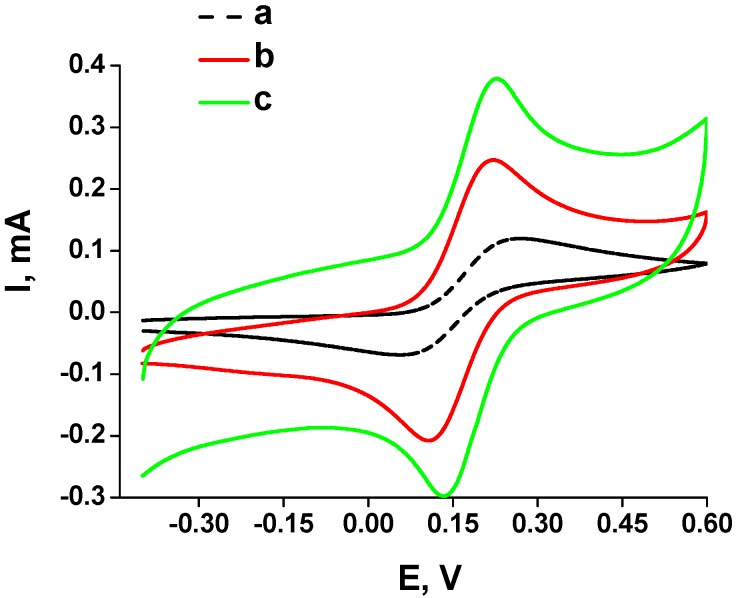
Cyclic voltammograms of the non-modified DRP-C220AT “DropSens” gold planar electrode (**a**); after modification by Pt-1 (**b**); andafter modification by Pt-2 (**c**) layers. Measurement conditions: frame scanned from −0.4 V to 0.6 V vs. Ag/AgCl; scan rate of 100 mV·s^−1^ in the electrolyte solution containing 10 mM K_4_Fe(CN)_6_ and 100 mM KCl.

The detailed analysis of electrochemical characteristics of the non-modified planar electrode and the planar electrodes modified by platinum was performed by cyclic voltamperometry in electrochemical cells with an electrolyte solution of K_4_Fe(CN)_6_ in KCl (Figure 4). As can be seen from Figure 4a, modification of the gold planar electrode by platinum significantly increased the electroconductivity of the working electrode (curves b and c) when compared with the non-modified electrode (curve a). Conductivity of the Pt-1-modified electrode increased 2.5-fold and that of the Pt-2-modified electrode increased more than four-fold compared to the non-modified electrode. The observed phenomenon of improving the electrochemical parameters of chemoelectrodes is expected to significantly increase the sensitivity of prototypes of FA-selective chemosensors.

### 2.3. Characterization of the FA-Sensitive Chemosensors

**Figure 5:**
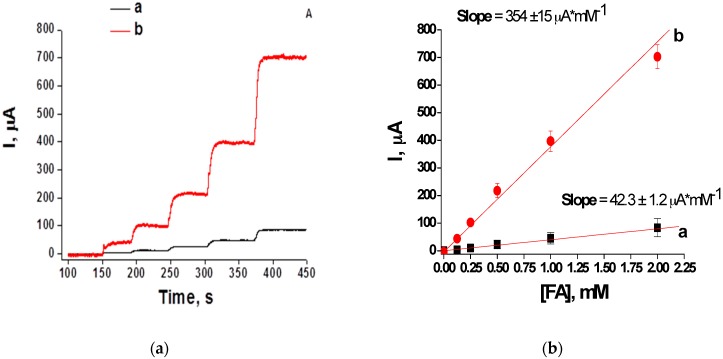
(**a**) Chronoamperometric response and (**b**) calibration curves in the linear frame upon FA addition of the chemosensors based on Pt-1 (**a**) and Pt-2 electrodes (**b**). Conditions: working potential 0.0 V vs. Ag/AgCl, working area 12.56 mm^2^, 20 mM PB, pH 7.0 under continuous stirring at room temperature.

The Pt-modified electrodes were used for construction of FA-selective chemosensors. For this purpose, chronoamperometric analysis of the Pt-1 and Pt-2 electrodes was performed upon addition of increasing concentrations of FA. The sensor output was measured (Figure 5a) and used for building calibration curves for each of the electrodes (Figure 5b, curves a and b). Both sensors exhibited a linear response up to 2 mM of FA. However, the chemosensor based on the Pt-2 electrode showed much higher sensitivity than the Pt-1 electrode. As can be seen from Figure 5, the Pt-2 sensor generated a current of 704 µA upon the addition of 2 mM FA, whereas the Pt-1 sensor generated a maximal output of only 84 µA under the same conditions (Figure 5a). The sensitivity of both chemosensors can be characterized as a specific response calculated from slopes of the calibration curves presented in Figure 5b. The sensitivity values of the constructed chemosensors were very high: 3400 A·M^−1^·m^−2^ for the Pt-1-based chemosensor and 28180 A·M^−1^·m^−2^ for the Pt-2-based electrode. It can be assumed that preparation of the chemosensor using the cyclic voltammetry technique enabled a more than eight-fold increase in sensitivity compared to the chemosensor produced electrochemically [20]. The detection limit for the Pt-2-based electrode determined from a chronoamperometric curve is 0.05 mM for FA (Figure 5a).

**Figure 6:**
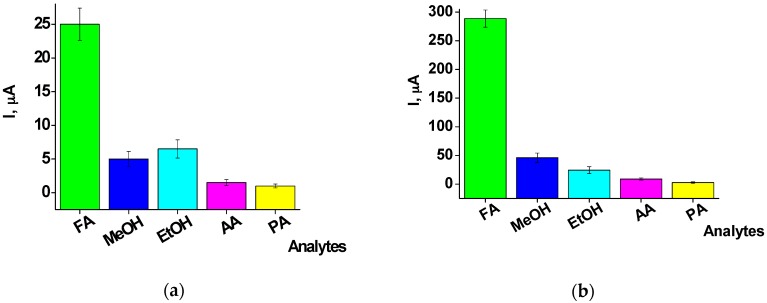
Analysis of the selectivity of (**a**) Pt-1 and (**b**) Pt-2 modified electrodes to various analytes at 1 mM concentration. Conditions: working potential 0.0 V vs. Ag/AgCl, 20 mM PB, PH 7.0 under continuous stirring at room temperature. Abbreviations: FA: formaldehyde; MeOH: methanol; EtOH: ethanol; AA: acetaldehyde; PA: propylaldehyde.

The most important characteristic of chemosensors is their selectivity to the target analyte. The response of both chemoelectrodes to several additional compounds which were applied at the same molar concentration was therefore tested. These compounds included methanol, ethanol, acetaldehyde, and propylaldehyde. The results presented in Figure 6 show that both chemosensors exhibited a high sensitivity only to FA, whereas responses to the otheranalytes were significantly lower. The sensitivity of the Pt-1 and Pt-2 electrodes for methanol was 20.0% and 16.6% of the response for FA, respectively (Figure 6a). For ethanol, the respective values were 26% and 8.3%, for acetaldehyde the values were 6% and 3%, and for propylaldehyde they were 2% and 1% (Figure 6b). It should be noted that the selectivity of the Pt-2 chemosensor towards FA exceeded that of the Pt-1 electrode in all cases.

The stability of the Pt-based chemosensors over time was examined. Both electrodes were found to remain absolutely stable over one year of storage in the dark at room temperature and can be reused over hundred times without any measurable worsening of electrochemical properties.

The developed chemosensors were used for analysis of the FA content in samples of the commercial preparations “Formalin” and “Sanodez Forte”. According to the manufacturers, the FA content in “Formalin” is 13.0 M and in “Sadonez Forte” it is 2.6 M. These samples were analyzed using the Pt-2-based chemosensor compared to several other chemical and enzymatic methods previously used by us (Table 1).

A standard multiple additions method for FA analysis by the chemosensor showed that the FA concentration in “Formalin” and in “Sanodez Forte” is 13.64 and 2.65 М, respectively. The obtained results correlate well with chemical and enzymatic spectrophotometric approaches (Table 1). A high (0.7 < *R* < 1) and significant (*p*-value < 0.001) correlation was found between the results of the FA-analysis using the chemosensor and the FA-content declared by the manufacturers.

The Pt-2 chemosensor shows better characteristics in FA monitoring compared to a majority of sensors constructed by others on the basis of various metals. A number of electrocatalysts have been developed and found appropriate for FA determination. However, in most cases the tests were performed in the presence of individually-taken FA concentrations and without any specification for a range of linear responses to variations in the FA concentration. This relates to platinum-based catalysts, such as polycrystalline platinum alone and containing electrodeposited ruthenium [42], platinum nanoparticles prepared on the surface of glassy carbon electrodes [27], and platinum nanoparticles deposited onto poly(o-methoxyaniline)-multiwalled carbon nanotubes under galvanostatic conditions [34], which were tested in the presence of 0.1 M, 0.32 M, and 1 M concentrations of FA, respectively. Since the experiments were performed at high FA concentrations, nothing can be concluded about the sensitivity of the catalysts and their potential applications as sensors for FA. A similar situation is observed in the case of electrocatalysts built on the basis of palladium: a copper-palladium electrode [43] and hollow porous palladium nanoparticles [44] were found to exhibit catalytic activity for electrochemical oxidation of FA. However, the tests were carried out only at 30 mM and 1 M FA, respectively, and no indication regarding the sensitivity and linear concentration range of the electrodes was reported. It should be mentioned that most of these electrocatalysts showed high stability after multiple uses and maintained an activity close to the initial activity after 50 [44], and even 1800 [34] cycles.

On the other hand, several authors constructed electrodes based on nanoparticles of noble and other metals and proved they could gain linear signals in response to variations in the FA concentration. A carbon paste electrode modified with nanoporous cobalt-nickel phosphate with dispersed nickel ions gained a linear signal at 3–15 mM of FA and showed good stability, retaining 94% of its activity after one month and 87% after three months [45]. A palladium-graphene electrochemical sensor developed by Qiao et al. [46] demonstrated a very low FA detection limit and high sensitivity (3467 A·M^−1^·m^−2^), but was characterized by a linear response in a very narrow range of 7.75 to 62 μM of FA. An electrode based on highly-dispersed platinum nanoparticles deposited electrochemically on graphene [31] showed a linear response up to 2 mM of FA, with a detection limit of 0.04 mM and a sensitivity of 0.0162 mA·mM^−1^ with a working electrode area of 7.07 mm^2^, which corresponds to 2290 A·M^−1^·m^−2^. The electrode retained 90% of its activity after 10 days of daily measurements. An electrochemical sensor on the basis of iron and platinum core-shell nanoparticles prepared on a carbon support was found suitable for determination of hydrogen peroxide, glucose and FA [41], and in the latter case showed a wide linear response range of 12.5 μM to 15.4 mM and a very low sensitivity of 117.5 A·M^−1^·m^−2^. Multipurpose applicability of this sensor seems to be advantageous over other types of sensors, but this feature actually constitutes a restriction for practical use due to its low selectivity.

The Pt-2 chemosensor constructed in the present work is characterized by a wide (0.02–2 mM) linear response range, by uniquely high sensitivity (28180 A·M^−1^·m^−2^), by good selectivity, and outstanding stability, which is very important for commercialization of the sensor. These characteristics, along with a simple preparation procedure, open prospects for implementation and practical application of this sensor for monitoring and accurate detection of FA in aqueous systems.

## 3. Materials and Methods

### 3.1. Materials

Ethanol absolute, acetaldehyde, potassium chloride, and hexacyanidoferrate(III) were purchased from Sigma Aldrich Chemie (Steinheim, Germany); hexachloroplatinum(IV)-acid hexahydrate, methanol, and propionaldehyde were obtained from Merck-Schuchardt (Hohenbrunn, Germany).

All chemicals were of analytical reagent grade and all solutions were prepared using HPLC-grade water. One molar FA solution was prepared by hydrolysis of 300 mg of paraformaldehyde in 10 mL of water by heating the suspension in a sealed ampoule at 105 °C for 6 h.

Tested real samples included the following preparations: “Formalin” (produced by “SferaSim”, Lviv, Ukraine) and “Sanodez Forte” (produced by “DezoMark”, Novoyavorivsk, Ukraine).

### 3.2. Scanning Electron Microscopy and X-ray Microanalysis

A scanning electron microscope (SEM-microanalyser REMMA-102-02, Sumy, Ukraine) was used for morphological characterization of the electrodes’ planar surfaces. A special cover film was formed on the samples with a Butvar solution B-98 (Sigma, St. Louis, MO, USA) in 1.5% chloroform using an ultrasound method. The distance from the last lens of the microscope to the sample (WD) ranged from 17.1 to 21.7 mm. The accelerator voltage was in the range from 20 to 40 kV. Zooms were from 2500× to 10,000×.

### 3.3. Preparation and Evaluation of the Chemosensors

The properties of the FA-sensitive amperometric chemosensors were evaluated by means of constant-potential amperometry in a three-electrode configuration using commercial 4 mm gold planar electrodes DRP-C220AT for repeated use fromDropSens (Llanera, Asturias, Spain). Amperometric measurements were carried out using a potentiostat CHI 1200A (IJ Cambria Scientific, Burry Port, UK) connected to a personal computer and performed in a batch mode under continuous stirring in a standard 20 mL electrochemical cell at room temperature. After 2 min of stabilizing the background current, the experiments were started by the addition of sample aliquots. During the course of the experiments, the modified electrodes were stored in air at room temperature.

All real samples were diluted using 0.05 mM sodium phosphate buffer, pH 8.0. The dilution factors for “Sanodez Forte” and “Formalin” were 2500 and 4000, and 6000 and 8000, respectively.

All experiments were carried out independently in triplicate and the reported results are the average of three replicate experiments. Statistical data evaluations were calculated using Origin 7.5 (OriginLab Corp., Northampton, MA, USA) and Microsoft Excel.

## 4. Conclusions

A new amperometric chemosensor based on a platinized gold electrode for accurate formaldehyde (FA) analysis was described. The platinization of the surface of 4 mm gold planar electrodes was performed electrochemically by electrolysis (Pt-1) and by cyclic voltamperometry (Pt-2). The structural modification of working electrodes by the two types of Pt layers was characterized using scanning electron microscopy and X-ray spectral analysis. A significant increase in electrochemical conductivity of the Pt-modified working electrodes was demonstrated compared with a non-modified electrode. The sensitivity of the Pt-2-based chemosensor for FA is uniquely high (28180 A·M^−1^·m^−2^), and is 79-fold higher than the sensitivity of an earlier FA-selective biosensor which we developed based on using formaldehyde dehydrogenase [25], and 8.1-fold higher compared to the chemosensor described by Qiao et al. [46]. Exploiting a low working potential (0.0 V vs. Ag/AgCl) enabled an increase in the selectivity of the chemosensor to the target analyte. The storage and operational stability of the Pt-based chemosensors was a very high, as proven after over one year of storage in the dark at room temperature and reusing them more than one hundred times.

The FA-selective chemosensor was tested on real samples of commercial preparations manufactured in Ukraine: “Formalin” and “Sanodez Forte”. A very high correlation was shown between the results obtained with the chemosensor and by chemical methods and enzymatic approaches (*R*=0.998–0.999). The developed prototype of a FA-selective amperometric chemosensor can be widely used in industry and research, as well as for environmental control.

## Figures and Tables

**Table 1:** The results of FA analysis of commercial preparations using the Pt-2-modified electrode chemosensor compared with chemical and enzymatic methods.

Product	FA Concentration Determined by Various Methods, M					
	Biosensor	Chemical (Spectrophotometric)			Declared by Producer	Chemosensor
	FdDH-Based [25]	Chromotropic [10]	MBTH [10,11]	Purpald [12]	-	Current Study
“Formalin”	13.5 ± 2.1	14.0 ± 2.4	12.6 ± 2.1	12.9 ± 1.9	13.0 ± 1.5	13.6 ± 1.8
“Sanodez Forte”	3.2 ± 0.6	3.6 ± 0.9	3.6 ± 0.8	3.3 ± 0.5	2.6 ± 0.5	2.7 ± 0.48
